# BambooGDB: a bamboo genome database with functional annotation and an analysis platform

**DOI:** 10.1093/database/bau006

**Published:** 2014-03-05

**Authors:** Hansheng Zhao, Zhenhua Peng, Benhua Fei, Lubin Li, Tao Hu, Zhimin Gao, Zehui Jiang

**Affiliations:** ^1^State Forestry Administration Key Open Laboratory on the Science and Technology of Bamboo and Rattan, International Center for Bamboo and Rattan, Beijing 100102, China, ^2^State key laboratory of tree genetics and breeding, Research Institute of Forestry, Chinese Academy of Forestry, Beijing 100091, China and ^3^Key Laboratory of Tree Breeding and Cultivation, State Forestry Administration, Research Institute of Forestry, Chinese Academy of Forestry, Beijing 100091, China

## Abstract

Bamboo, as one of the most important non-timber forest products and fastest-growing plants in the world, represents the only major lineage of grasses that is native to forests. Recent success on the first high-quality draft genome sequence of moso bamboo (*Phyllostachys edulis*) provides new insights on bamboo genetics and evolution. To further extend our understanding on bamboo genome and facilitate future studies on the basis of previous achievements, here we have developed BambooGDB, a bamboo genome database with functional annotation and analysis platform. The *de novo* sequencing data, together with the full-length complementary DNA and RNA-seq data of moso bamboo composed the main contents of this database. Based on these sequence data, a comprehensively functional annotation for bamboo genome was made. Besides, an analytical platform composed of comparative genomic analysis, protein–protein interactions network, pathway analysis and visualization of genomic data was also constructed. As discovery tools to understand and identify biological mechanisms of bamboo, the platform can be used as a systematic framework for helping and designing experiments for further validation. Moreover, diverse and powerful search tools and a convenient browser were incorporated to facilitate the navigation of these data. As far as we know, this is the first genome database for bamboo. Through integrating high-throughput sequencing data, a full functional annotation and several analysis modules, BambooGDB aims to provide worldwide researchers with a central genomic resource and an extensible analysis platform for bamboo genome. BambooGDB is freely available at http://www.bamboogdb.org/.

**Database URL**: http://www.bamboogdb.org

## Introduction

As a tribe of flowering and evergreen perennial monocot classified in the subfamily Bambusoideae within the grass family Poaceae that includes rice, maize, wheat and other cereals (1), bamboo is one of the most important non-timber forest resources in the world (2). Because of having high strength-to-weight ratio, like natural woody, bamboo is natural composite material, which is useful for making construction material, paper pulp and furniture (3). Recent data make clear that ∼2.5 billion people in the world depending on bamboo for their daily lives, and the international trade volume on bamboo amounts to 2.5 billion US dollars per year (4). Moreover, bamboo grows widely in tropical and subtropical of Asia, Africa, northern Australia and Latin America, extending as far north as the southern United States and as far south as Patagonia. About 1000 species of woody bamboo are widely distributed all over the world, among which ∼100 species were used commercially. Moso bamboo (*Phyllostachys edulis*) is one of the most important economic bamboo species with many advantages such as fast growth rate, high yield, extensive use, short crucial period formation and strong regeneration capacity (5).

During the past several decades, many studies have been carried out on the bamboo using various biotechnologies, such as biochemical, physiological, cytogenetic and genomic methods, mainly including chloroplast genome sequencing, identification of syntenic genes between bamboo and other grass and phylogenetic analysis of Bambusoideae subspecies (6–10). Additionally, a series of studies on moso bamboo were performed, including the first high-quality genome sequence by *de novo* sequencing (11), deep RNA sequencing (RNA-seq) for seven samples in different tissues (11), and the cloning and sequencing of 10 608 putative full-length complementary DNA (cDNA) (3). However, data from different researches are scattered in publications, and the lack of a systematic review and analytical platform of the currently available data and knowledge has remained a longstanding challenge for genetic and genomic of bamboo.

Here, we report BambooGDB, a bamboo genome database with functional annotation and analysis platform, mainly based on the *de novo* sequencing data of moso bamboo. In addition, the RNA-seq and full-length cDNA data were also included in BambooGDB to enrich the contents of this database. On the basis of these large-scale sequencing data, a comprehensive annotation for bamboo genome was made, including basic annotation for bamboo genes, RNAs, proteins and heterozygous single nucleotide polymorphisms (SNPs), as well as functional annotations, such as gene ontology, Kyoto Encyclopedia of Genes and Genomes (KEGG) pathway, orthologs and protein–protein interaction (PPI). Besides, an analytical platform composed of comparative genomic analysis, PPI network, pathway analysis and visualization of genomic data were constructed to extend our understanding of the bamboo genome and to help researchers to design experiments for further validation. Furthermore, to facilitate the navigation of these data, diverse and powerful search tools and a convenient browser were also incorporated in BambooGDB. Through integrating high-throughput sequencing data, a full annotation and several analysis modules, BambooGDB was designed to a central genomic resource and an extensible analysis platform for bamboo genome to facilitate future studies and help reveal the genomic features of bamboo and other related plants.

## Data Content

There are three types of data included in BambooGDB: (i) the high-quality genome sequence data of moso bamboo (11), (ii) the genome-wide full-length cDNA data of moso bamboo (3) and (iii) the deeply sequenced RNA-seq data for seven samples in different tissues of moso bamboo (11). It is worth mentioning that all of the aforementioned data of moso bamboo were mainly obtained from International Center for Bamboo and Rattan, which has long been dedicating to the biological research on bamboo. In addition, to carry out comparative genomic analysis between bamboo and other plants, we also collect the whole genome data of two model plants and five bamboo-related species, which includes *Arabidopsis thaliana* (12)*, Oryza sativa* (13), *Brachypodium distachyon* (14), *Panicum virgatum* (sequenced by the US Department of Energy Joint Genome Institute), *Sorghum bicolor* (15), *Setaria italica* (sequenced by the US Department of Energy Joint Genome Institute) and *Zea mays* (16). Currently, there are >33 000 annotated bamboo genes in this database. The summary of data content in BambooGDB is shown in Table 1.

**Table 1.** BambooGDB data content and statistics as of 12 October 2013

Data set	Data type	Data statistics
Basic data content	DNA/Protein	
	Genes	31 987
	Expressed genes (FPKM^a^ ≥ 1)	28 576
	MicroRNA target genes	161
	Proteins	31 987
	RNA	
	tRNAs	1167
	MicroRNAs	86
	Variant	
	Heterozygous SNPs^b^	2 009 487
Annotation	DNA/Protein	
	Pfam-A accessions	21 645
	COGs^c^ accessions	14 049
	InterPro accessions	66 567
	PANTHER^d^ accessions	38 868
	EC^e^ numbers	752
	Ortholog groups	9856
	Structure feature	
	Conserved domain models	852 248
	Conserved sites	36 731
	Pathway/Network	
	GO^f^ accessions	37 188
	KO^g^ accessions	3714
	Metabolic pathway	
	Proteins	3946
	Pathway maps	191
	PPI	
	Proteins	2202
	Interactions	34 169
	Comparative genomics	
	Best *Arabidopsis* hits	16 383
	Best rice hits	21 849

^a^FPKM: Fragments per kilobase of transcript per million mapped reads.

^b^Heterozygous SNPs: heterozygous single nucleotide polymorphisms.

^c^COGs: Clusters of orthologous group.

^d^PANTHER: Protein ANalysis THrough Evolutionary Relationships (one classification system of protein).

^e^EC: Enzyme commission.

^f^GO: Gene ontology.

^g^KO: KEGG orthology.

## Genome Annotation and Data Analyses

**Figure 1.**
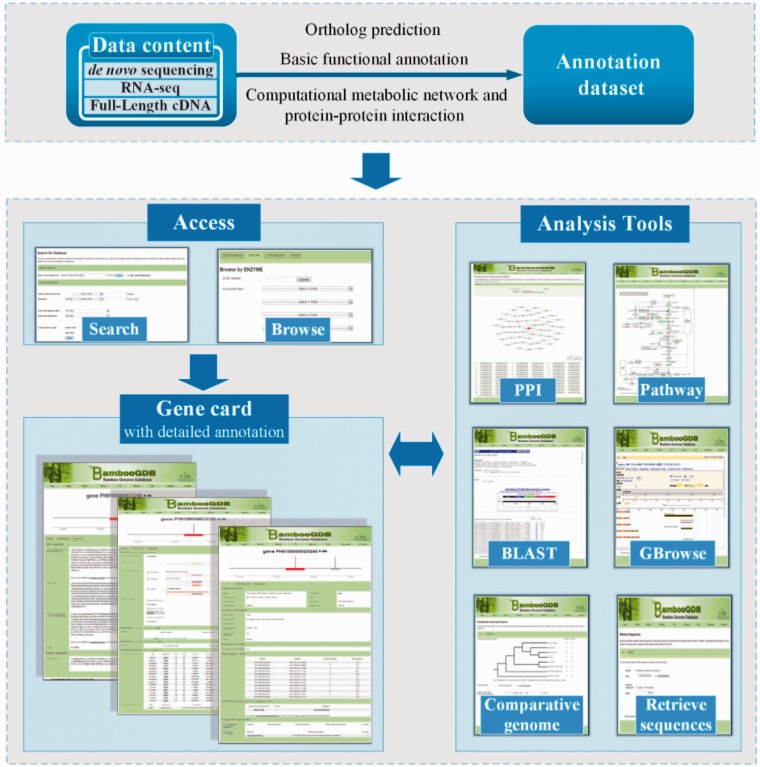
Screenshot showing interrelation of data and tools housed in BambooGDB. Users access the data through search and browse function. In addition, all data and tools incorporated in BambooGDB are cross-linked.

Based on the large-scale sequencing data, a series of annotation under multilevel was conducted, including fundamental annotation of function such as motif, domain and structure analysis, comparative analysis among bamboo-related species, metabolic pathway network analysis and PPIs network analysis (Figure 1). As shown in Table 1, the data statistics of BambooGDB dated 12 October 2013. Moreover, the detailed information was concisely presented in three aspects as follows:

### Basic functional annotation

As a central part of BambooGDB, genomic functional annotation plays a fundamental role in genomic studies. To obtain comprehensive genomic functional information, a series of annotation and analysis work was performed with five following aspects. First, the prediction of gene function motifs and domains was performed by InterProScan (Release 5 Candidate 6) software (17) against InterPro database (18), which has integrated together predictive information about proteins function from a number of partner resources and provided an overview of the function and domain of protein. Therefore, several kinds of valuable classifications were obtained in result list of InterProScan, such as PRINTS (19), Pfam-A (20), Gene3D (21), PANTHER (22), InterPro (17) and Gene Ontology (23). Second, clusters of orthologous group (COGs) were predicted by BLASTP (24) against COG database (25) in NCBI under *E*-value 1e^−^^6^. Third, based on previous study (26), full-length cDNA sequences of moso bamboo were mapped to its genome using BLAT (27). Fourth, structure features of protein were predicted by Batch CD-Search web services in NCBI (28). Finally, the bamboo gene models were aligned to entries of sorghum, rice and maize from the KEGG database (29) by BLASTP under *E*-value 1e^−^^10^ to find the best hit for each gene in the similar pathway.

### Computational metabolic network and PPI

Computational network analysis is a kind of efficient method and tool for investigating the features that identify the topology of a metabolic network and the interaction of relative compounds. As one of the important components in computational network analysis, computational metabolic network and PPIs network for bamboo were analyzed and then implemented in BambooGDB.

As an important database and platform for study of molecular interactions, the KEGG database provides a reference knowledge base for connecting genomes to the biological functions. Therefore, on the basis of the KEGG database, proteins of moso bamboo were annotated with the KEGG orthology (KO) by using the best hit information. In addition, the graphical display used in KEGG Automatic Annotation Server (30) can help user to visually understand the characteristics of metabolic pathways. Finally, there were 3946 proteins and 191 pathway maps in computational metabolic network of moso bamboo.

Computational identification of PPIs network in moso bamboo can provide a new insight into cellular functions of proteins. The computational process was briefly introduced as follows. First, linkages with protein sequences of moso bamboo and UniProt database (release 201308) (31) were established by BLASTP comparison with the following criteria. (i) *E* < 1.0 × 10^−^^10^, (ii) sequences identify >40% and (iii) aligned sequence length coverage >40%. In this study, the aligned sequence length coverage was strictly defined as the aligned sequence length of the query without gaps divided by the whole sequence length of the query. Then, using UniProt access numbers as input, PPIs of moso bamboo were computed on protein interaction network analysis platform, which included a database of unified PPI data from six manually curated public database (32,33). Therefore, the final PPIs network in moso bamboo contained 2202 proteins with 34 169 interactions.

### The analysis of comparative genomes

Orthologs are homologs separated by speciation events, and prediction of orthologs is then becoming much more important with the rapid progress in genome sequencing (34,35). Some of grasses family (Poaceae), including *B. distachyon*, *P. virgatum*, *O. sativa*, *S. bicolor*, *S. italica*, *Z. mays* and *P. edulis*, are more essential than any other groups of plants for food and potential renewable energy. Owing to the number of genome sequences increasing much more rapidly than any other plant family, the grass family is an ideal group for comparative studies on orthologs analysis. In BambooGDB, prediction of orthologs among bamboo-related species was performed on the whole genome scale using OrthoMCL algorithm, which provided a scalable approach to constructing orthologous groups across multiple eukaryotic taxa basing a Markov cluster algorithm to group orthologs (36). Moreover, to well complement text-based searches for similar genes with sequence-based methods, an interface for more flexibly viewing BLAST tool was developed. The BLAST tool in BambooGDB contained genome, coding sequence and protein sequence information for *P. edulis*, *O. sativa* and *A. thaliana*, respectively.

## Data Usage and Analysis Tools

### Powerful search tools and a convenient browser

There are two basic ways for users to access data stored in BambooGDB (Figure 1): search and browse. Besides the simple keyword search, BambooGDB has also offered advanced search with a Boolean search to allow users to specify and combine query options by functional characteristics, such as COG accession, InterPro accession/description, Gene Ontology accession/term, EC number and pathway information. BambooGDB has provided not only a powerful search engine but also a user-friendly interface to browse various data and data connections. Moreover, the previous and valuable results for genomic analysis were also displayed such as RNA-seq and predicted microRNA data.

### Metabolic network analysis

The result of metabolic network predicted by KEGG Automatic Annotation Server was disposed in the pathway module of BambooGDB. The proteins of moso bamboo were assigned by KO, and automatically generated KEGG pathways were contained in BambooGDB as well. User can browse detailed information of protein when the mouse hovers over the icon of EC number in pathway maps and clicks an interested protein then to access particular gene card.

### PPI analysis

The computational PPIs data of moso bamboo were integrated in the PPI module of BambooGDB. Users can submit a protein name as a query protein and then an image and a table will be generated by predicting the interacted partners of the query protein. The image is produced by Cytoscape (37), which is an open source bioinformatics software platform emphasized on providing analyses of visualizing networks. In addition, PPI also supports users to input a group of protein to explore the interactions among them.

### Comparative genomic analysis based on bamboo-related species

In the orthologs groups’ module of gene card, the results of predicted orthologs were demonstrated. Meanwhile, in the comparative genome search available from tools, we provide searching function to find orthologous genes that are present in one set of genomes and absent in another set among bamboo-related species. Moreover, sequences from various species also could be aligned by BLAST tool, which was incorporated in BambooGDB.

### GBrowse

GBrowse is a combination of database and interactive web pages for manipulating and visualizing annotations on genomes. Entries from the various type data are marked in different colors in the browser. As an important and efficient visualization module, GBrowse (38) was incorporated in BambooGDB to facilitate viewing different types of factors (gene, CDS, messenger RNA, full-length cDNA, heterozygous SNPs and RNA-seq) simultaneously in the context of genomic regions. Users can also connect to the detailed feature page of corresponding entries from the browser.

## Application

BambooGDB is a novel resource of functional annotation for bamboo genome and analytical platform to facilitate studies about genetic and genomic of bamboo. By managing the integration of genome data of moso bamboo, data connection and analysis tools, researchers may start from a single gene or functional term of interest to acquire a relatively comprehensive knowledge of functional annotation in different research levels, such as expression and functional regulation.

**Figure 2.**
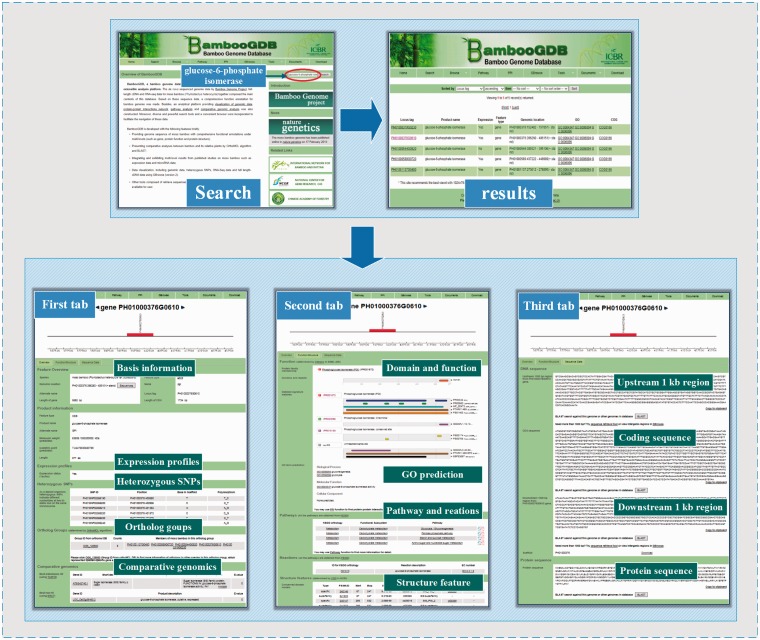
One example of the application of BambooGDB for browsing and searching information for bamboo research.

For basic research, BambooGDB provided a fully functional annotation for moso bamboo. For example, as the shown in Figure 2, some researchers might be interested in ‘glucose-6-phosphate isomerase’ and tend to find and understand the genetic information of this protein in bamboo. To achieve this, first, by using search function in BambooGDB, we searched ‘glucose-6-phosphate isomerase’ as search content quickly at home page, then results ultimately will be linked to result page. There are a total of five results in BambooGDB. Second, click the locus (‘PH01000376G0610’), as an interested gene, and then enter gene card, which includes a graphical view of the local genomic environments and three tabs for details. In the first tab, there were fundamental annotation information such as gene/protein name, length and location, expression profiles, heterozygous SNPs, ortholog group and best hits with *Arabidopsis* or rice. Based on the information of ‘orthologs groups’, we found the aforementioned result of search together belong to ‘OG5_126980’ group. For detailed information of this group, we can click ‘OG5_126980’ to browse further information in database of orthoMCL DB. Moreover, according to the information of comparative genomics, the best hit for locus PH01000376G0610 is *AT5g42740.1* and LOC_Os03g564860.2, in the genome of *Arabidopsis* and rice, respectively. In the second tab, comprehensive functional and structural feature of the homologous glucose-6-phosphate isomerase in moso bamboo was highlighted. For instance, the information of domain was visually displayed by comparative functional domain, such as the sequence of PH01000376G0610 mainly matched the domain of glucose-6-phosphate isomerase. Similarly, the prediction of GO term showed PH01000376G0610 belong to ‘glucose-6-phosphate isomerase activity’ (GO: 0004347) in the category of molecular function. In the pathway part, it was demonstrated that PH01000376G0610 participated in four carbohydrate metabolisms (glycolysis/gluconeogenesis, pentose phosphate pathway, starch and sucrose metabolism as well as amino sugar and nucleotide sugar metabolism), and contained the following information: KO (K01810), reaction description (glucose-6-phosphate isomerase) and EC number (EC: 5.3.1.9). In the third tab, the sequence information of locus PH01000376G0610 was presented, including CDS sequence, upstream/downstream 1000-bp region away from locus PH01000376G0610 and its protein sequence. BambooGDB also provided the module of ‘retrieve sequences’, which will display and download specific sequences in the genome of moso bamboo. Moreover, the aforementioned information of sequence can be conveniently downloaded by clipboard function implemented in BambooGDB. Additionally, further information on each functional characteristics can be accessed via hyperlinks to external corresponding database.

**Figure 3.**
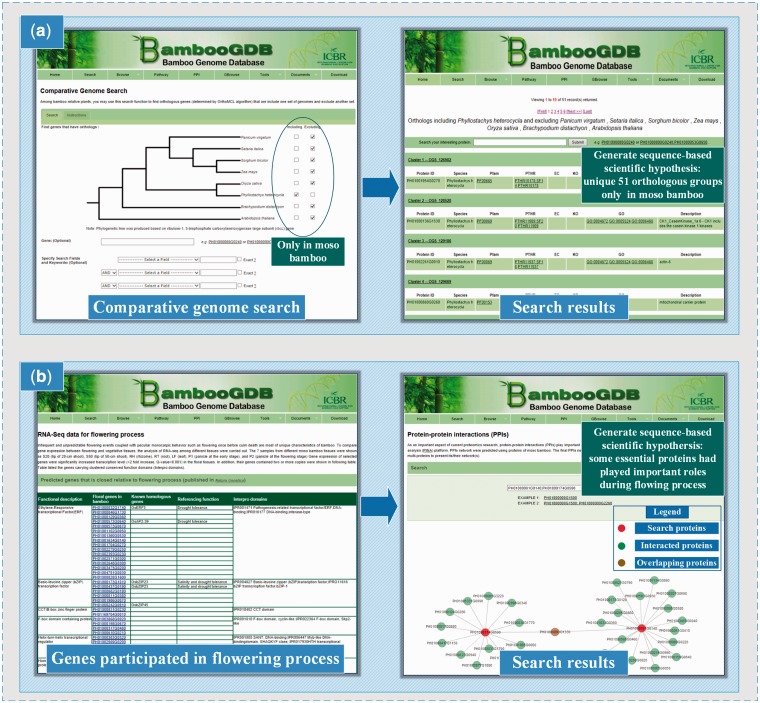
Another example of the application of BambooGDB in deriving data-based new hypothesis for bamboo research. (a) An application example for studying on bamboo special characteristics; (b) An application example for studying on bamboo flowering mechanism and protein-protein interactions.

Another example of application is on study of special characteristics on bamboo. It is generally known that the moso bamboo has outstanding and unique features, For example, moso bamboo has a rapid speed of growth with an eventual height of >10 m during a short period of 2–4 months (39). To help researchers conveniently uncover special features of bamboo distinguished from other plants with genome sequenced, ‘comparative genome search’ was implemented in BambooGDB to get an overview of unique orthologs in bamboo. In the first place, accessing to ‘comparative genome search’ page from the module of ‘tools’ (Figure 3a), researchers attempt to look for orthologous proteins that were only present in moso bamboo but were absent in *P*. *virgatum*, *S*. *italica*, *S*. *bicolor*, *Z*. *mays*, *O*. *sativa*, *B*. *distachyon* and *A*. *thaliana*. In the next place, on the basis of selecting ‘*P*. *edulis’* from ‘including’ box and selecting ‘*P*. *virgatum’*, ‘*S*. *italica’*, ‘*S*. *bicolor’*, ‘*Z*. *mays’*, ‘*O*. *sativa’*, ‘*B*. *distachyon’* as well as ‘*A*. *thaliana’* from ‘excluding’ box, the result was shown that a total of 52 proteins in 51 ortholog clusters were predictably unique in moso bamboo. Additionally, it is another striking features of bamboo that the vegetative phase can last up to one hundred or more years before flowering and then followed death after flowering (40). As researchers tend to identify, and focus on, the genes or proteins during flowering stage, the gene study of flowering stage in moso bamboo becomes increasingly important in uncovering flowering mechanism and developing effective method for breeding. To facilitate the discovery of potential genes for flowering is a valuable application of BambooGDB in research. First, ‘RNA-Seq data for flowering process’ from the module of ‘Browse’ in BambooGDB collected a total of 115 genes, which were identified as the flowering genes in bamboo not only by analyzing gene expression between flowering and vegetative tissues in bamboo but also by aligning sequence with known flowering genes in rice (11). Second, to provide new clues on candidate proteins, BambooGDB provides an analysis tool of ‘protein–protein interactions (PPIs)’ to assign biological functions of the uncharacterized proteins. For example, we searched locus ‘PH01000081G0140’, as one of flowering proteins was focused, in search box of the module of PPIs. The results shown proteins interacted with ‘PH01000081G0140’ may be the mainly participants in flowering process. Meanwhile, PPIs also provided the function of multiprotein search. For instance, we search the two flowering proteins ‘PH01000081G0140’ and ‘PH01000174G0590’, the result then demonstrated not only proteins interacted with each of search proteins but also proteins interacted with both of search proteins as well. As the shown in Figure 3b, ‘PH01000093G1330’, generally has collaborative or similar functions in biology processes (41), is an important participant interacted with both of ‘PH01000081G0140’ and ‘PH01000174G0590’. Therefore, aforementioned novel predictions by using BambooGDB will help drive new finding to accelerate our knowledge on bamboo mechanism.

## System Design and Implementation

The front-end web application was developed in a Centos Linux 6.4 environment using a combination of Java EE 1.6 (Java Platform Enterprise Edition), Freemarker 2.3.20, Perl 5, Apache Web Server 2.0 and Apache Tomcat 7.0.40. In back-end part, all of the data in BambooGDB were stored and managed in MySQL relational databases. Moreover, to accelerate the performance of GBrowse displays, a large amount of trace data after being converted into genome feature format (GFF-Version 3) were imported into MySQL databases as well. GBrowse was built following the configuration files provided by its developer (http://gmod.org/wiki/GBrowse_Configuration_HOWTO).

## Discussion and Future Development

As the first genome database with functional annotation for bamboo, BambooGDB aims to act as not only an integrated genomic resource special for bamboo but also a flexible computational platform for the genetic studies of bamboo in future. In addition, after firstly moso bamboo *de novo* sequencing genome published, the number of genome resequencing, RNA-seq, rare variants, epigenetics and other omics studies for bamboo is expected to keep increasing especially with the development of sequencing technologies in the next few years. Therefore, BambooGDB will be periodically updated to ensure a most up-to-date follow up of the omics research progress of bamboo. Meanwhile, manual curation of literature for genetics of bamboo will be carried out to fulfill the increasing research demands in addressing the genetic complexity of bamboo. The scope of BambooGDB will be expanded to integrate newly generated data. We hope our continuous efforts will help to better understand the genome for bamboo.
